# Supplementary material for: Comparison of OneChoice AI-based clinical decision support recommendations with infectious disease specialists and non-specialists for bacteremia treatment in Lima, Peru
Source: PLoS One. 2026 Apr 17;21(4):e0331266. doi: 10.1371/journal.pone.0331266 (PMC13089868; doi:10.1371/journal.pone.0331266)
Supplement: S1 Appendix — https://doi.org/10.6084/m9.figshare.31281913. (PDF) [file pone.0331266.s001.pdf]

XYZ DIAGNOSTICS

Antimicrobial Stewardship: Molecular Lab Result

Powered by ARKSTONE

**Report ID**	XXXXXXX	**Patient**	XXXXX, XXXXX	**DOB**	XX/XX/XXXX	**Collected**	XX/XX/XXXX
**Specimen Type**	Blood	**Facility**	XXXXXXXXX	**Resulted**	XX/XX/XXXX	**Received**	XX/XX/XXXX

Infection Complexity **ARK**SCORE™

LO 2 HI

Organisms Detected

Common pathogens in bold

- **Escherichia coli**

Resistance Detected

Extended-Spectrum Beta-Lactamase

Antimicrobial Resistance **ARK**SCORE™

LO 3 HI

No Allergies Reported

OneChoice Drug Info

Ertapenem

Dose Adj ☑ Renal ☐ Hepatic

Interactions Valproic acid

Avg Price $ $ $ $

Adverse Reaction **ARK**SCORE™

LO 3 HI

ONECHOICE® PLUS

Dose adjustments, other drug options, references, translations and support

ONECHOICE®

Ertapenem 1 gm IV Q24H x 7-14 days for possible bacteremia

Alternative Treatment Options with Adverse Reaction Arkscore™

- **Gentamicin** ARKSCORE 5 5-7 mg/kg IV/IM Q24H (peak and trough adjusted) x 7-14 days
- **Meropenem** ARKSCORE 3 1-2 gm IV Q8H x 7-14 days
- **Cipro** ARKSCORE 5 400 mg IV Q12H x 7-14 days

° See additional dosing info on OneChoice Plus. Gentamicin efficacy against E. coli is uncertain.

Why is this the OneChoice?

E. coli can be pathogenic when found in blood samples. Resistance genes were detected which may limit available treatment options. The recommended treatment is optimal as it targets all the concerning microbes. ‡

When should this be treated?

Bacteria in blood should always be treated when contamination is not of concern. The source of the bacteremia should be determined in order to assure the infection is treated adequately, as antibiotics may need to be tailored specifically to the source of the infection. For many microbes, de-escalation to oral antibiotics is not possible. The duration of treatment depends on the source of infection but is generally 7-14 days at a minimum and when complicated can extend for several weeks. ‡

Are there any special considerations?

As ESBL resistance is on the CDC threat list, tracking and monitoring may be indicated if possible. ESBL can be associated with other resistance genes. Antibiotics should therefore be used with caution as drug failure is possible. Blood cultures are typically positive in the presence of infection. However, false positives may occur due to contamination. Resistance detected may only affect certain microbes, and in some cases, none at all. ‡

Infection Control Precautions: ☑ Standard ☑ Contact

* Dosing and duration of treatment based on adult patient, with no medical history, normal BMI, renal and hepatic functions, and minimal time required to treat simple infections. Treatment is directed at common pathogens noted above, and the most commonly associated antibiotic resistance based on genes detected. Resistance is variable and drug failure is possible. Additional microbiology workup and treatment modification may be needed. Visit OneChoice Plus for expanded information.

‡ For education purposes only. This is not a diagnosis. Clinical correlation and physician judgment required when making diagnosis or treatment decisions. Recommendations based on lab results, and limited to specimen source, organisms, resistance, allergies, and ICD10 codes. Patient has not been examined nor their medical history reviewed.

Copyright 2026 Arkstone Medical Solutions. OneChoice, MedsMatrix, and ArkScore are based on patent pending methods and algorithms. Learn more at arkstone.ai/report
