# Supplementary material for: Comparison of OneChoice AI-based clinical decision support recommendations with infectious disease specialists and non-specialists for bacteremia treatment in Lima, Peru
Source: PLoS One. 2026 Apr 17;21(4):e0331266. doi: 10.1371/journal.pone.0331266 (PMC13089868; doi:10.1371/journal.pone.0331266)
Supplement: S3 Appendix — https://doi.org/10.6084/m9.figshare.31281952. (PDF) [file pone.0331266.s003.pdf]

Correlation between the clinical decision-based system (Onechoice®) with recommendations from physicians of various specialties for the choice of antimicrobials in bloodstream infections

Greetings, colleague. Thank you very much for participating in this study. This analysis compares pharmacotherapy decision-making based on healthcare professionals' experience with alternatives provided by a Human-in-the-Loop Machine Learning system developed by Arkstone Medical Solution. This survey is aimed at healthcare professionals with a medical specialty, so no additional personal data beyond what is asked for is necessary.

The goal is to select the best therapeutic option, including dosage and duration of the selected antimicrobial, based on the results provided. Please consider yourself in a scenario where only the pharmacotherapy available in your country can be employed. Some cases include conditional events like recent antibiotic use, allergies, and other particular conditions. This information will appear in the question. Avoid using guidelines or virtual tools that might assist you in your decision-making process.

To start the survey, please read the informed consent and select the ACCEPT option to continue:

- Accept ☐
- Reject ☐

Informed Consent

Dear Doctor,

We cordially invite you to participate in the research project entitled:
"Accuracy of therapeutic recommendations from a clinical decision support system: Onechoice® report based on phenotypic results compared with infectious diseases specialists and non-specialists in the treatment of urinary tract infections diagnosed in Lima, Peru."
This study aims to evaluate the agreement between the recommendations generated by the Onechoice® system and the clinical decisions made by physicians specialized in Infectious Diseases compared with physicians from other specialties in the management of urinary tract infections.

What does your participation involve?
Your participation will consist of completing a digital survey (Google Forms) based on fictional clinical cases. The survey includes a total of 14 questions distributed across 7 clinical cases. The collected data will be analyzed to determine the accuracy of the recommendations provided by the Onechoice® system in comparison with the clinical decisions made by specialists.

Confidentiality and Data Handling
All information obtained will be handled with strict confidentiality. The results will be used exclusively for academic and research purposes. None of the collected data will allow the identification of participants, thus guaranteeing their anonymity.

Benefits and Risks
By participating, you will contribute to the development of tools that may improve clinical decision-making in the treatment of severe infections. This study does not pose any risk to your well-being and does not require any procedures beyond your usual clinical practice.

Voluntary Participation
Your participation is completely voluntary. You may choose not to participate or withdraw your consent at any time without affecting your professional relationship with the institution.
If you have any questions or require further information, please do not hesitate to contact the principal investigator:

Juan Carlos Gómez de la Torre Pretell
Phone: +51 965378787
Email: jgomez@labroe.com

We sincerely appreciate your collaboration in this important research project.

Sincerely,
Juan Carlos Gómez de la Torre Pretell

Correlation between the clinical decision-based system (Onechoice®) with recommendations from physicians of various specialties for the choice of antimicrobials in bloodstream infections

Basic information

Age (in numbers) *

Tu respuesta

Country where you work *

PERU

Enter the registration number that accredits your professional medical title, according to the country where you practice your profession: *

Tu respuesta

Years practicing the clinical specialty *

- 1 - 5 years
- 6 - 10 years
- 11 - 15 years
- 16 - 20 years
- More than 20 years

Clinical specialty *

- Infectology
- Internal medicine
- Rheumatology
- Endocrinology
- Pulmonology
- Geriatrics
- Cardiology
- Otro:

Case 31 (AS_5207_301958193): A 79-year-old female patient, hospitalized (from a rural area, self-sufficient, with no apparent comorbidities), who presented fever during the last five days, nonspecific abdominal pain, and vomiting. The treating physician requested laboratory analyses, including blood samples for culture. The incubator's alarm triggered sixteen hours after extracting her sample, and the laboratory technicians performed a FilmArray® Blood panel. Two hours later, the attached image was reported. Based on this result, what therapeutic option would you recommend? Consider dosage, interval, frequency, and duration (assuming the bacteremia is uncomplicated). The patient has no allergies to antimicrobials. **Your recommendation should align with the Principles of Antimicrobial stewardship and current guidelines.** * 5 puntos

Organisms

Streptococcus sp ✓

Undetected Organisms

- Acinetobacter ×
- Bacteroides fragilis ×
- Candida albicans ×
- Candida auris ×
- Candida glabrata ×
- Candida krusei ×
- Candida parapsilosis ×
- Candida tropicalis ×
- Cryptococcus neoformans/gattii ×
- Enterobacter cloacae ×
- Enterobacterales ×
- Enterococcus faecalis ×
- Enterococcus faecium ×
- Escherichia coli ×
- Haemophilus influenzae ×
- Klebsiella aerogenes ×
- Klebsiella oxytoca ×
- Klebsiella pneumoniae ×
- Listeria monocytogenes ×

Resistance Genes

Undetected Genes

Carbapenem	NDM	×
Carbapenem	KPC	×
Carbapenem	IMP	×
Carbapenem	VIM	×
Colistin	mcr-1	×
Vancomycin	VanA/B	×
Methicillin	mecA/C	×
Methicillin	mecA & MRE...	×
Extended-Spectrum Beta-Lactamase	BLEE CTX-M	×
Carbapenem	OXA-48 Lik...	×

- Neisseria meningitidis ×
- Proteus sp ×
- Pseudomonas aeruginosa ×
- Salmonella spp ×
- Serratia marcescens ×
- Staphylococcus aureus ×
- Staphylococcus epidermidis ×
- Staphylococcus lugdunensis ×
- Staphylococcus sp ×
- Stenotrophomonas maltophilia ×
- Streptococcus agalactiae ×
- Streptococcus pneumoniae ×
- Streptococcus pyogenes ×

- ◯ Aztreonam 500mg IV every 12h x 14d
- ◯ Penicillin G 24 MU in continuous infusion IV in 24h x 7-14d
- ◯ Ciprofloxacin 200mg IV every 12h x 14d
- ◯ Piperacillin/tazobactam 2.75mg IV every 8h x 7-14d

If your first recommended option is unavailable, which of the following antimicrobial options would you recommend as an alternative? * 5 puntos

- ◯ Imipenem 1g IV every 6h x 7-14d
- ◯ Ciprofloxacin 200mg IV every 12h x 7-14d
- ◯ Trimethoprim/sulfamethoxazole 160/800mg IV every 24h x 10d
- ◯ Ceftriaxone 1-2 g IV every 12 h x 7-14d

Regarding case 31: Blood cultures yielded results after 3 days, revealing the isolation of Streptococcus equi subsp. zooepidemicus using MALDI-TOF, with no apparent resistance genes identified. The attached antibiogram is shown, and the patient remains clinically stable. Based on this information, what therapeutic recommendation would you suggest, considering dosage, interval, frequency, and duration? Please assume the bacteremia is uncomplicated. * 5 puntos

Streptococcus equi spp zooepidemicus

SENSITIVITIES	
Amox/Clav	S
Azithromycin	S
Cefixime	S
Cefotaxime	S
Ceftriaxone	S
Chloramphenicol	S
Cipro	S
Clindamycin	S
Erythromycin	S
Levofloxacin	S
Linezolid	S
Moxifloxacin	S
Penicillin G	S
Teicoplanin	S
Tetracycline	S
TMP-SMX	R
Vancomycin IV	S

- Cefepime 1g IV every 8h x 14d
- Ciprofloxacin 400mg IV every 8h x 14d
- Meropenem 2g IV every 6h x 14d
- Penicillin G 24 MU in continuous IV infusion in 24h x 7-14d

[illegible]

In addition, what therapeutic alternatives would you consider if the first option cannot be administered for a number of reasons? Keep in mind, your recommendation should align with the principles of antimicrobial stewardship and current guidelines. * 5 puntos

- Ciprofloxacin 200mg IV every 12h x 7-14d
- Ampicillin 1-2g IV every 4-6h x 7-14d
- Trimethoprim/sulfamethoxazole 160/800mg IV every 6h x 14d
- Imipenem 500mg IV every 6h x 14d
